# Plaie pénétrante du genou compliquée d'une section totale du tendon patellaire et d'une rupture du rétinaculum patellaire médial

**DOI:** 10.11604/pamj.2015.20.327.6635

**Published:** 2015-04-06

**Authors:** Youness Sasbou, Omar Mourafiq

**Affiliations:** 1 Service de Chirurgie Orthopédique II, Hôpital Militaire d'Instruction Mohammed V, Rabat, Maroc

**Keywords:** Plaie, tendon patellaire, rétinaculum, wound, patellar tendon, retinaculum

## Image en medicine

Les plaies du genou avec une section du tendon patellaire et une rupture du rétinaculum patellaire médial sont des lésions rares, et peu rapportés dans la littérature. Le diagnostic est essentiellement clinique et les principaux signes sont l'ascension et la latéralisation de la patella et surtout le déficit de l'extension active du genou. Les sections du tendon patellaire nécessitent une réparation immédiate afin de rétablir l'appareil extenseur et de permettre une récupération fonctionnelle précoce et les ruptures du rétinaculum patellaire médial nécessitent une suture pour éviter l'instabilité patellaire. Nous rapportons un cas rare d'une plaie pénétrante du genou cachant une section complète du tendon patellaire associée à une rupture du rétinaculum patellaire médial. Il s'agit d'un patient âgé de 25 ans, victime d'une agression par coup de couteau entrainant une plaie pénétrante de la face antérieure du genou droit (A). L'examen physique montre un déficit de l'extension active du genou droit. La radiographie standard de face du genou (B) a montré une ascension de la patella. Le patient a bénéficié d'un parage puis exploration chirurgicale de la plaie qui a objectivé une section totale du tendon patellaire et une rupture du rétinaculum patellaire médial (C). La réparation a été réalisée par une suture du rétinaculum médial puis d'une suture tendineuse directe termino-terminale du tendon patellaire par un fil à résorption lente 2mm renforcée par un surjet, suivie d'une suture du péri-tendon et protégée par un cadrage tendineuse au fil d'acier 16/10 (D) enlevée à la 6^ème^ semaine postopératoire avec une bonne évolution à 6 mois de recul.

**Figure 1:**
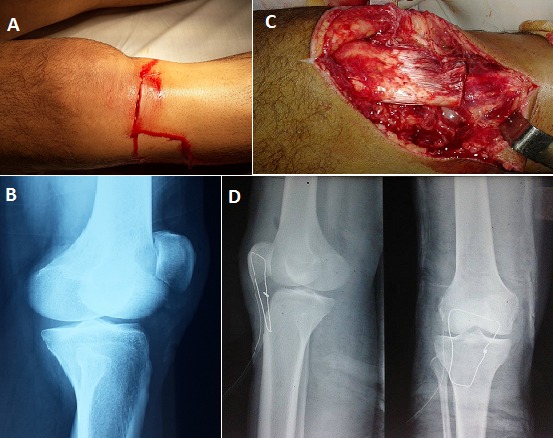
(A): image montrant la plaie pénétrante du genou; (B): radiographie standard du genou en incidence de profil montrant l'ascension de la patella; (C): vue per-opératoire montrant la section complète du tendon patellaire; (D): radiographie standard du genou en incidence de face et de profil montrant le cadrage de protection de la suture tendineuse

